# Fusion of multiple heterogeneous networks for predicting circRNA-disease associations

**DOI:** 10.1038/s41598-019-45954-x

**Published:** 2019-07-03

**Authors:** Lei Deng, Wei Zhang, Yechuan Shi, Yongjun Tang

**Affiliations:** 1 School of Computer Science and Engineering, Central South University, Changsha, 410075 China; 2 Department of Pediatrics, Xiangya Hospital, Central South University, Changsha, 410008 China

**Keywords:** Data integration, Computational models

## Abstract

Circular RNAs (circRNAs) are a newly identified type of non-coding RNA (ncRNA) that plays crucial roles in many cellular processes and human diseases, and are potential disease biomarkers and therapeutic targets in human diseases. However, experimentally verified circRNA-disease associations are very rare. Hence, developing an accurate and efficient method to predict the association between circRNA and disease may be beneficial to disease prevention, diagnosis, and treatment. Here, we propose a computational method named KATZCPDA, which is based on the KATZ method and the integrations among circRNAs, proteins, and diseases to predict circRNA-disease associations. KATZCPDA not only verifies existing circRNA-disease associations but also predicts unknown associations. As demonstrated by leave-one-out and 10-fold cross-validation, KATZCPDA achieves AUC values of 0.959 and 0.958, respectively. The performance of KATZCPDA was substantially higher than those of previously developed network-based methods. To further demonstrate the effectiveness of KATZCPDA, we apply KATZCPDA to predict the associated circRNAs of Colorectal cancer, glioma, breast cancer, and Tuberculosis. The results illustrated that the predicted circRNA-disease associations could rank the top 10 of the experimentally verified associations.

## Introduction

Circular RNA (circRNA) is a class of non-coding RNA recently discovered. Unlike linear RNA, circRNA forms a continuous cycle of covalent closures and is highly represented in the eukaryotic transcriptome. Previous research has found thousands of prototype circRNAs in human, mouse and nematode cells^1–4^. As the report goes, circular RNA in higher organisms were produced by reverse splicing events and synthesized from all regions of the genome, mainly from exons, and a few from antisense, intergenic, intragenic and intron regions^5^.

The expression level of circRNA is low, and thus, it was initially thought that circRNA was a by-product of splice-mediated splicing errors or an intermediate that escaped from the intron lariat^6–8^. Therefore, circRNA received little attention in the past. However, with the development of high-throughput sequencing technology and computational analysis techniques, thousands of circRNAs have been discovered in many species ranging from archaea to humans, and the expression level of some circRNAs was ten-fold higher than those obtained from the standard linear transcription of homologous genes^3,4,9–13^.

A large number of studies have revealed many circRNA functions, such as serving as scaffolds in the assembly of protein complexes, isolating proteins from their natural subcellular localization, regulating the expression of parental genes, modulating alternative splicing and RNA-protein interactions, and functioning as microRNA (miRNA) sponges^10,14–18^. In addition to their potential function such as significant regulators of gene expression, circRNAs were reported to be related to many different human diseases, including neurodegenerative disorders and cerebrovascular diseases. In particular, experiments have shown that many circRNAs are closely related to cancer^19–21^, and some experimental evidence demonstrated that circRNA plays an essential role in atherosclerotic vascular diseases, prion diseases and cancers of the nervous system, especially exhibiting abnormal expression level in colorectal cancer (CRC) and pancreatic ductal adenocarcinoma (PDAC). In this way, the circRNA could act as a biomarker for the diagnosis and prediction of some diseases in the future.

Several circRNA related resource databases have recently been established. The circBase database^22^ combines data from several circRNAs, including circular RNA IDs, genomic coordinates, and optimal transcripts, into a standardized database. The CircNet database^23^ provides a new circRNA identification tool that offers annotation of genomic circRNA isoforms and circRNA subtype sequences by integrating circRNA-miRNA-mRNA regulatory networks. The Tissue-Specific CircRNA Database (TSCD)^24^ provides circRNAs obtained from cancer cells with four algorithms, and the corresponding features of circRNAs, such as cancer-specific circRNAs (CS-circRNAs), RBP-binding sites in CS-circRNAs, cancer-specific alternative splicing associated with CS-circRNAs, miRNA target sites in CS-circRNAs, and possible open reading frames in CS-circRNAs. The CircInteractome database^25^ maps RNA-binding protein (RBP) sites on circRNAs, which can be used to search for potential interactions between circRNAs and RBPs or miRNAs, and for potential internal ribosomal entry sites. SomamiR2.0^26^ provides target sites for mutations in tumor cells or miRNA cells, while this kind of mutation might alter the interaction of miRNAs with circRNAs. Circ2Traits^27^ first validated the interaction between circRNAs and miRNAs, calculating those circRNAs which are likely to be associated with a disease. This database then identified the Argonaute (Ago) interaction site on circRNAs. The Cancer-Specific CircRNA Database (CSCD)^28^ provides circRNAs obtained from cancer cells using four algorithms and the corresponding features of circRNAs, such as cancer-specific circRNAs (CS-circRNAs), RBP-binding sites in CS-circRNAs, cancer-specific alternative splicing associated with CS-circRNAs, miRNA target sites in CS-circRNAs, and potential open reading frames in CS-circRNAs. The CircR2Disease database^29^ provides experimentally demonstrated circRNA-disease associations and includes detailed information on the associations, such as the disease names, circRNA names, expression patterns, detection methods and simple descriptions. However, the circRNA-disease associations supported by experimental evidence remain relatively rare.

**Figure 1:**
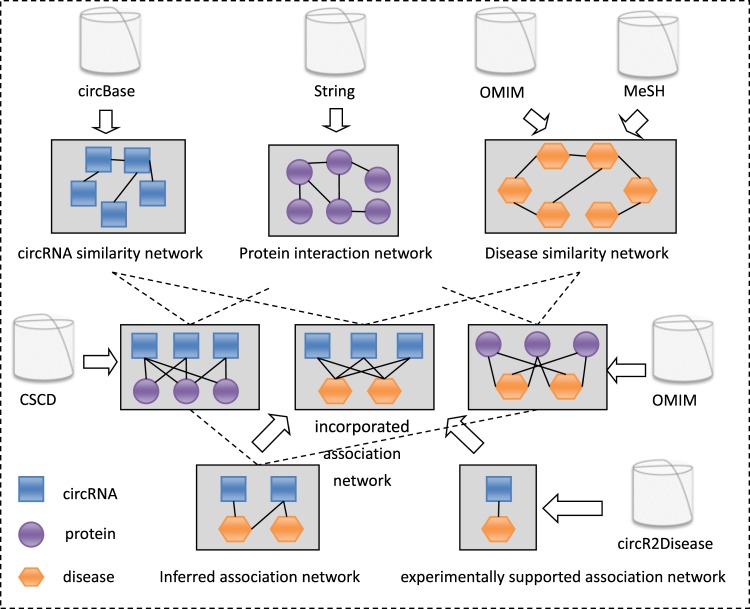
Flowchart for constructing an incorporated circRNA-disease association network.

In this study, based on the “gilt-by-association” (GBA) principle, which states that biological entities having the same or related behaviour tend to be associated^30^, we assumed that circRNAs associated with the same protein tend to be associated with protein-related diseases. Based on existing resources and previous studies on endogenous non-coding RNAs with disease associations and the GBA principle, we proposed a computational model named KATZCPDA to predict circRNA-disease associations. We first obtained an inferred circRNA-disease association network from a known circRNA-protein association network and a protein-disease association network. Then we gained a known circRNA-disease association network from the circR2Disease database and finally integrated the inferred network with the known network to achieve an incorporated circRNA-disease association network. The KATZCPDA model thus predicts potential circRNA-disease associations using the KATZ method integrated with the combined circRNA-disease association network, disease similarity network, and circRNA similarity network (Fig. 1). Based on the circRNA-disease associations supported by experiments included in the CircR2Disease database^29^, we analysed KATZCPDA using leave-one-out cross validation (LOOCV) and 10-fold cross-validation. The results showed that KATZCPDA achieved a significantly higher performance than the existing methods.

## Results

### Datasets

#### circRNA similarity matrix

We obtained the circRNA expression profiling data from the work of Peng *et al*.^31^, which include expression profiles of 2,895 human circRNAs. The circRNA similarity matrix *CS* was built by computing Pearson’s correlation coefficient (PCC) between the expression profiles of each pair of circRNAs. If the PCC score between circRNA *i* and circRNA *j* is lower than the threshold, we set *CS(i, j)* to be 0. Otherwise, we updated *CS(i, j)* to be 1.

#### Matrix of circRNA-protein associations

The circRNA-protein association dataset was downloaded and compiled from the CSCD database^28^ (http://gb.whu.edu.cn/CSCD/), which deposit more than 270,000 cancer-specific circRNAs. CSCD also include circRNA binding proteins (RBPs). Based on the circRNA-protein association dataset, we used the adjacency matrix *CP* to describe the association network between circRNAs and proteins: if circRNA *i* is associated with protein *j*, *CP(i,j)* is set to be 1.

#### Matrix of protein-disease associations

The OMIM database^32^ (http://www.omim.org/downloads/) contains information on known Mendelian disorders and over 15,000 genes. Here, we choose to use the associations between proteins and phenotypes updated in October 2018. The adjacency matrix *PD* was used to indicate the functional similarity between proteins and diseases. If protein *i* is associated with disease *j*, *PD(i,j)* = 1; otherwise, *PD (i, j)* = 0.

#### Disease similarity matrix

The disease similarity matrix consists of integrated phenotypic information. Because the disease names in CircR2Disease^29^ are not standardized (the index is not corresponding to the standard database, such as ENSEMBL and RefSeq), we obtained disease-related indexes via manual matching. First, we collected all diseases from the confirmed circRNA-diease association to obtain the list of disease names and then manually searched for each disease in the OMIM database to obtain the closest correlation phenotype ID (In the OMIM database, a prefix of none, % or # usually means that the ID provides a phenotype description). To ensure the accuracy of the data, the diseases that failed to match the phenotype ID in the OMIM database and the corresponding circRNA-disease associations were removed. The disease similarity matrix was obtained using the text mining method developed by Driel *et al*.^33^, in which the entity *DS(i,j)* in the *ith* row and the *jth* column represents the disease similarity score between diseases *d(i)* and *d(j)*. According to Oron Vanunu *et al*.^34^, similarity scores greater than 0 and less than 0.3 are not informative, while similarity scores greater than 0.6 and less than 1 indicate informative similarity, illustrating a potential similarity between these two diseases. In this study, if the similarity score was less than the threshold 0.4, we replaced the similarity score with 0. If the similarity score was greater than the 0.4, we updated the similarity score to 1.

#### Matrix of circRNA-disease associations

Seven hundred forty circRNA-disease associations were downloaded from the CircR2Disease database^29^ (http://bioinfo.snnu.edu.cn/). We obtained a dataset of 263 high-quality circRNA-disease associations containing 222 circRNAs and 46 diseases. Since the experiment determined circRNA-disease associations in CircR2Disease is limited, we obtained an inferred circRNA-disease network by integrating the collected circRNA-protein associations and protein-disease associations. Based on the inferred circRNA-disease association network, we built an integrated circRNA-disease association network *G*_*mix*_ for the KATZCPDA computational model.

### Evaluation measures

In this section, we evaluated the performance of the proposed method through leave-one-out cross-validation (LOOCV) and 10-fold cross-validation. In the LOOCV, each circRNA-disease association was individually left out in turn to form the test set, and remaining disease-circRNA associations were used as to train the model. In the 10-fold cross-validation, we randomly divided the circRNA-disease associations into ten subsets. And then we left out one subset as the test set, using the remaining nine subsets to train the model.

With both LOOCV and 10-fold cross-validation, for each query (circRNA) node, its predicted association score with all target (disease) nodes can be obtained. We generated a plot of the ROC curves according to the false positive rate (FPR) and true positive rate (TPR) using each iteration for different thresholds. The simultaneous calculation of the area of the ROC curve yielded the AUC value that can be used to assess overall performance.

### Effects of inferred circRNA-disease associations

To demonstrate the effects of the inferred circRNA-disease associations established with protein information, we tested two different networks via LOOCV and 10-fold cross validation: (1) only circRNA disease associations with experimentally confirmed circRNA-disease association networks and (2) circRNA-disease association networks that contain both experimentally validated and inferred circRNA-disease associations (see the Results section for a description of the partial circRNA-disease association network).

**Figure 2:**
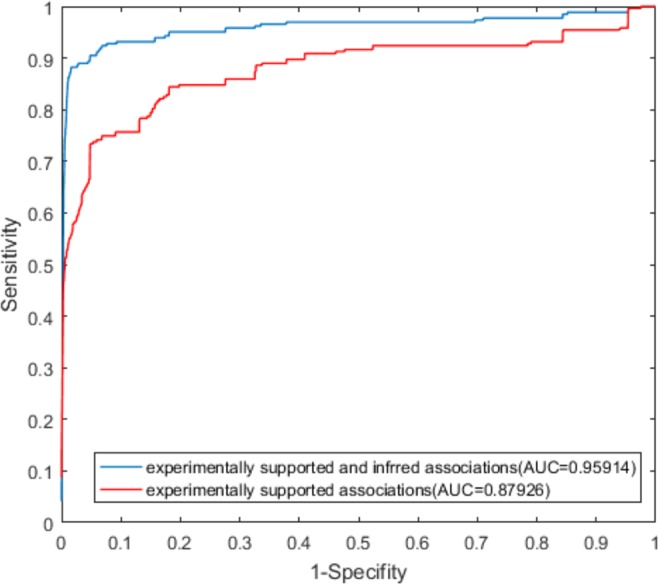
ROC curves from LOOCV using only experimentally validated associations and both experimentally validated and inferred associations.

**Figure 3:**
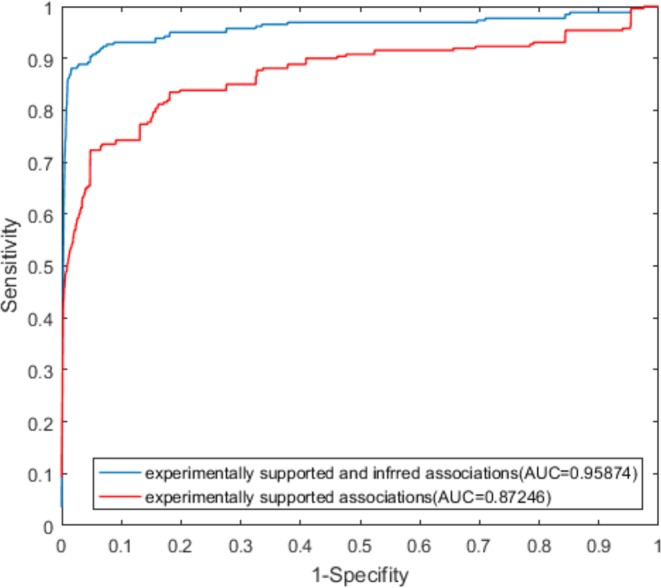
ROC curves from 10-fold cross validation using only experimentally validated associations and both experimentally validated and inferred associations.

As shown in Figs 2 and 3, the LOOCV and 10-fold cross-validation using both experimentally supported and inferred associations showed better performance than that established with only experimentally supported associations. In LOOCV, the use of both experimentally validated and inferred associations yielded an AUC value of 0.95914, and the use of only associations supported by experimental evidence yielded an AUC value of 0.87926. In 10-fold cross-validation, the AUC value obtained from both experimentally supported and inferred association was 0.95874, and that value obtained from the only associations supported by experimental evidence was 0.87246.

### Comparison with other methods

**Figure 4:**
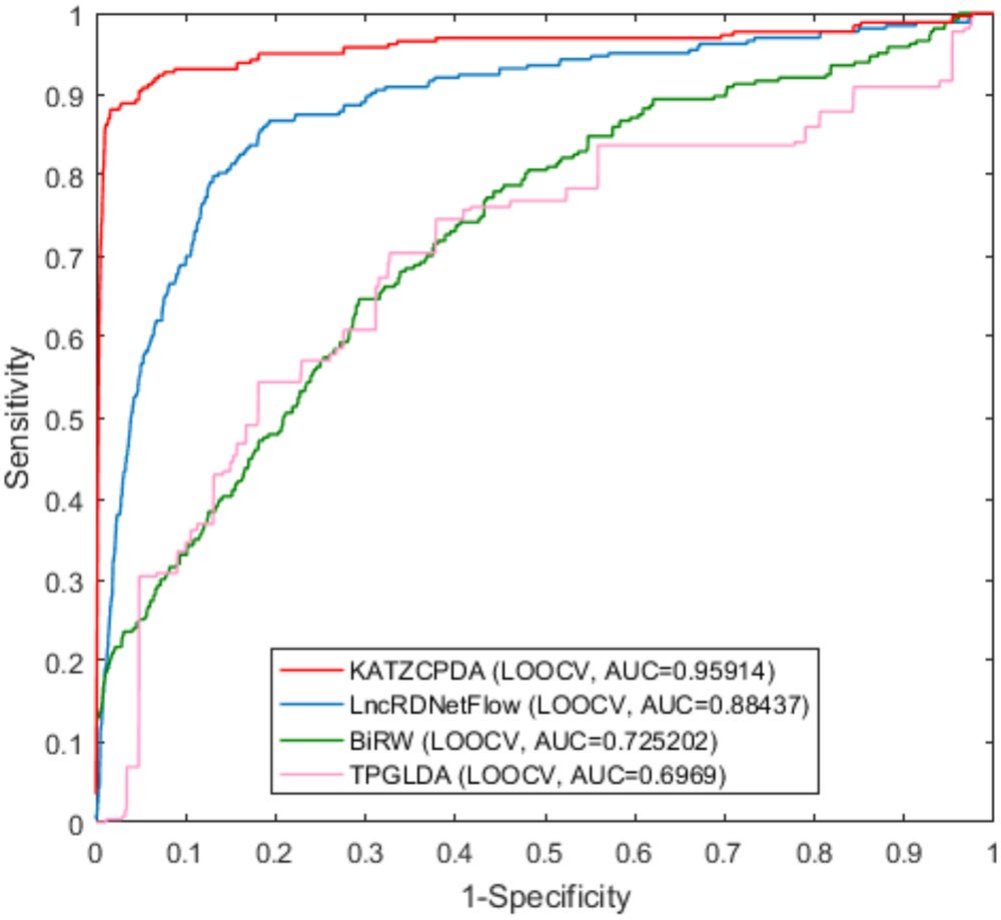
Comparison of the performances of KATZCPDA and other methods in terms of the ROC curve and AUC based on LOOCV.

**Figure 5:**
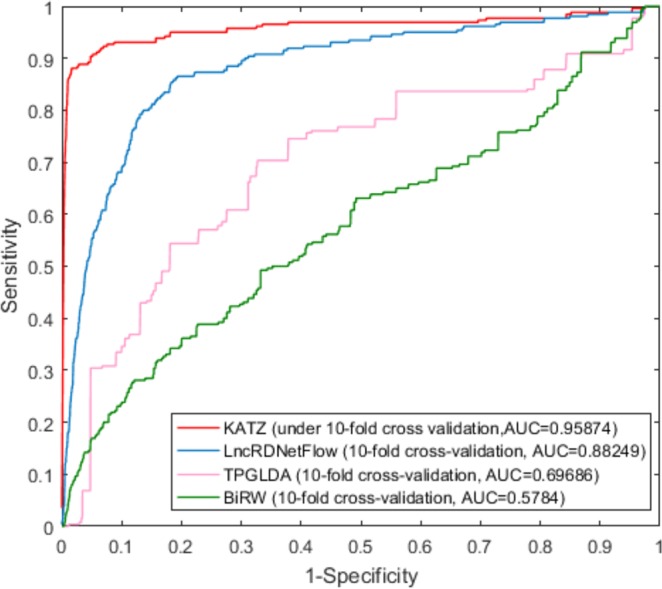
Comparisons of the performances of KATZCPDA and other methods in terms of ROC curve and AUC based on 10-fold cross-validation.

To further evaluate the performance of our approach, we compared KATZCPDA with three other predictive approaches (LncRDNetFlow^30^, TPGLDA^35^, and BiRW^36^), which can predict associations between various biological entities based on integrated network information. We trained and tested all the models on the same dataset. As it shown in Fig. 4, the AUC value for KATZCPDA obtained by LOOCV was 0.95914, and this value was significantly higher than the AUC values of the other three methods (LncRDNetFlow: 0.88437, TPGLDA: 0.6969 and BiRW: 0.725202). Similarly, as shown in Fig. 5, in the 10-fold cross-validation, the AUC value obtained for KATZCPD was 0.95874, which was also higher than the AUC values obtained for the other three methods (LncRDNetFlow: 0.88249, TPGLDA: 0.69686 and BiRW: 0.5784). Therefore, compared with BiRW, KATZCPDA is stable, as proved by both LOOCV and 10-fold cross-validation, because the deletion of the number of edges in the network substantially affects BiRW, i.e., the deletion of some edges in the 10-fold cross validation led to a noticeable decrease in the BiRW performance.

### Case studies

To further assess the validity of KATZCPDA, all circRNA-disease connections were utilized as training data for the models, and the diseases that were predicted to be associated with circRNAs were validated using the experimentally confirmed circRNA-disease associations in the CircR2Disease database. Here, we checked the circRNAs associated with three cancers and a diseases (colorectal cancer, glioma, breast cancer, and Tuberculosis), and Table 1 lists the corresponding rankings.

**Table 1:** Ranking of diseases among all diseases predicted to be associated with select circRNAs.

circRNAs	Diseases	KATZCPDA rank
hsa_circ_0000567	Colorectal cancer	4
hsa_circ_0008509	Colorectal cancer	4
hsa_circ_0007534	Colorectal cancer	6
hsa_circ_0007031	Colorectal cancer	6
hsa_circ_0000504	Colorectal cancer	6
hsa_circ_0000199	Glioma	3
hsa_circ_0005603	Glioma	4
hsa_circ_0006460	Glioma	5
hsa_circ_0004872	Glioma	5
hsa_circ_0008345	Glioma	7
hsa_circ_0006411	Glioma	7
hsa_circ_0011946	Breast cancer	6
hsa_circ_0001982	Breast cancer	5
hsa_circ_0002874	Breast cancer	6
hsa_circ_0085495	Breast cancer	6
hsa_circ_0001875	Breast cancer	5
hsa_circ_0000681	Tuberculosis	1
hsa_circ_0030045	Tuberculosis	2
hsa_circ_0030569	Tuberculosis	3
hsa_circ_0008797	Tuberculosis	3

Colon cancer is one of the most common diseases in the world, and even in developed countries, the mortality rate of colon cancer still remains high^37^. In China, the recent prevalence of colon cancer has risen due to unhealthy lifestyles^38^. Some studies have shown that colon cancer and circRNAs are closely related. Based on this conclusion, we predicted the associations of different circRNAs with colon cancer using KATZCPDA. As a result, colon cancer ranked high in the lists of diseases that were predicted to be associated with selected circRNAs. Among the diseases that were predicted to be associated to hsa_circ_0014717, hsa_circ_0000567, hsa_circ_0020397, hsa_circ_0007031, and hsa_circ_0007534, colon cancer ranked 4th, 4th, 4th, 4th, and 6th, respectively. The CircR2Disease database verified the associations between these circRNAs and colon cancer. For example, the database indicates that hsa_circ_0014717acts as a potential tumour suppressor that inhibits CRC growth, at least partly by upregulating p16 expression^39^.

Glioma is the most common primary mesenchymal tumour in the central nervous system and is the most common malignant tumour associated with morbidity and mortality. Patients with this cancer have poor prognosis because glioma is strongly invasive and aggressive. Previous studies have proved that circRNA dysregulation might be related to the occurrence and development of glioma and indicated that circRNAs can serve as prognostic biomarkers for glioma^40^. We analysed the relevant circRNAs using our KATZCPDA model to predict their associated diseases and found that gliomas ranked very high; specifically, glioma was ranked 5th, 5th, 7th, 5th, 7th, and 7th in the list of diseases associated with hsa_circ_0006460, hsa_circ_0005603, hsa_circ_0008345, hsa_circ_0004872, hsa_circ_0006411, and hsa_circ_0003586, respectively. Zhu *et al*.^41^ confirmed that hsa_circ_0006460 is related to gliomas. In addition, circBRAF (hsa_circ_0006460) is an independent biomarker for prognosticating good progression-free survival and overall survival in glioma patients^40^.

Breast cancer is the most common cancer among women worldwide. Epidemiological studies have shown that advanced age, oestrogen and progestin use, elderly primiparity, alcohol consumption and lack of physical exercise can increase the risk of breast cancer in women. In addition to genetic mutations, epigenetic mechanisms, including DNA histone modification, methylation and ncRNA, also play crucial roles in breast cancer^42^. circRNAs belonging to ncRNA are also believed to be potentially associated with breast cancer. We analysed the relevant circRNAs using the KATZCPDA calculation model and calculated their related diseases. Among these diseases, breast cancer was ranked at the top of the list of associated diseases. Among the illnesses associated with hsa_circ_0011946, hsa_circ_0001982, hsa_circ_0001785, hsa_circ_0001785, and hsa_circ_0002113, mammary gland cancer was ranked 6th, 6th, 6th, 6th, and 8th, respectively. As detailed in the database, hsa_circ_0001982 has been experimentally proved to be associated with breast cancer, and miR-143 has been demonstrated to be a target of hsa_circ_0001982 through a dual-luciferase reporter assay. In addition, loss-of-function and rescue experiments have indicated that hsa_circ_0001982 could knockdown and suppress breast cancer cells proliferation and invasion, also could induce apoptosis by targeting miR-143^43^.

Tuberculosis (TB) is a potentially severe infectious disease and is one of the significant threats to human health. Early correct diagnosis and fast curative treatment help prevent tuberculosis. Studies show that circRNA might serve as a potential new biomarker for tuberculosis infection^44^. We analyzed the relevant circRNAs using the KATZCPDA model and calculated the corresponding diseases. Among these predictions, tuberculosis ranks very high, even in some cases ranks the first. Some research conducted by Qian *et al*.^45^ showed that circRNAs such as hsa_circ_0000681 and hsa_circ_0008797 are closely related to tuberculosis.

## Discussion and Conclusion

Increasing lines of evidence show that circRNAs are closely related to many different diseases, such as Alzheimer’s disease, liver cancer and lung cancer. Some studies have explored the specific dysregulation of circRNA in infections and indicated that circRNA is a promising biomarker for diagnosis, treatment, and prognosis. Because novel experimental approaches have several limitations, models that integrate multiple biological datasets to infer circRNA-disease association can be used as supplementary tools for the detection of disease biomarkers. In this study, we integrated the known associations between circRNAs and proteins, proteins and diseases to infer circRNA-disease associations. Using the inferred circRNA-disease associations and the experimentally supported circRNA-disease associations as predictors, the KATZCPDA algorithm was then developed to predict circRNA-disease associations by integrating known biological information (circRNA similarity, disease similarities, protein-protein interactions, and the associations between these entities). Even in the absence of some associations, our method predicts new circRNA-disease associations successfully. In other words, when constructing a network of circRNA-disease associations, the bioinformatic analysis of the integrated protein information can infer potential information that cannot be obtained with only circRNA and disease information. In addition, the method is bidirectional because it can predict both circRNA-disease and disease-circRNA associations.

To verify the reliability of the predictive performance of KATZCPDA, we assessed different methods through LOOCV and 10-fold cross-validation using same datasets. The results showed that KATZCPDA has better performance than LncRDNetFlow, TPGLDA, and BiRW. The analysis of KATZCPDA for the prediction of the associations of circRNAs with colon cancer, glioma, breast cancer, and Tuberculosis revealed that the proposed method has excellent performance. Thus, KATZCPDA is likely to play an essential role in the identification of potential circRNA-disease associations in the future.

KATZCPDA can be improved in several aspects in the future. First, the diseases in the CircR2Disease database are not accurate. Although we obtained the most closely related OMIM ID from the OMIM database, a specific deviation in the disease similarity score might still exist. This requires discovering and integrating more reliable data. Second, the identification of circRNA similarity associations and the use of more effective methods to infer circRNA-disease associations can significantly improve the performance of the method. Third, the introduction of a higher number of intermediary entities to identify circRNA-disease associations is beneficial to increase the accuracy of the model prediction.

## Methods

Network inference techniques and machine learning approaches have been widely used in many classification fields^46–56^. In this study, we proposed a KATZ measure^57^ based approach (KATZCPDA) to predict unknown circRNA-disease associations by measuring the similarities between circRNAs of interest and diseases in the heterogeneous network. KATZ measurements can make successful predictions from social networks, disease-gene association networks, disease-lncRNA association networks, microbe-disease association networks, and disease-miRNA association networks^57–62^. KATZ is a graph-based calculation method that transforms the association prediction problem into the problem of calculating the similarity between nodes in a heterogeneous network. In the constructed global network, the prediction of the association between circRNA and disease nodes, is translated into the calculation of the number of walks and the range of walks connecting the corresponding circRNAs and diseases. The integration of the number of walks and length can yield the potential association probability of each circRNA-disease pair.

**Figure 6:**
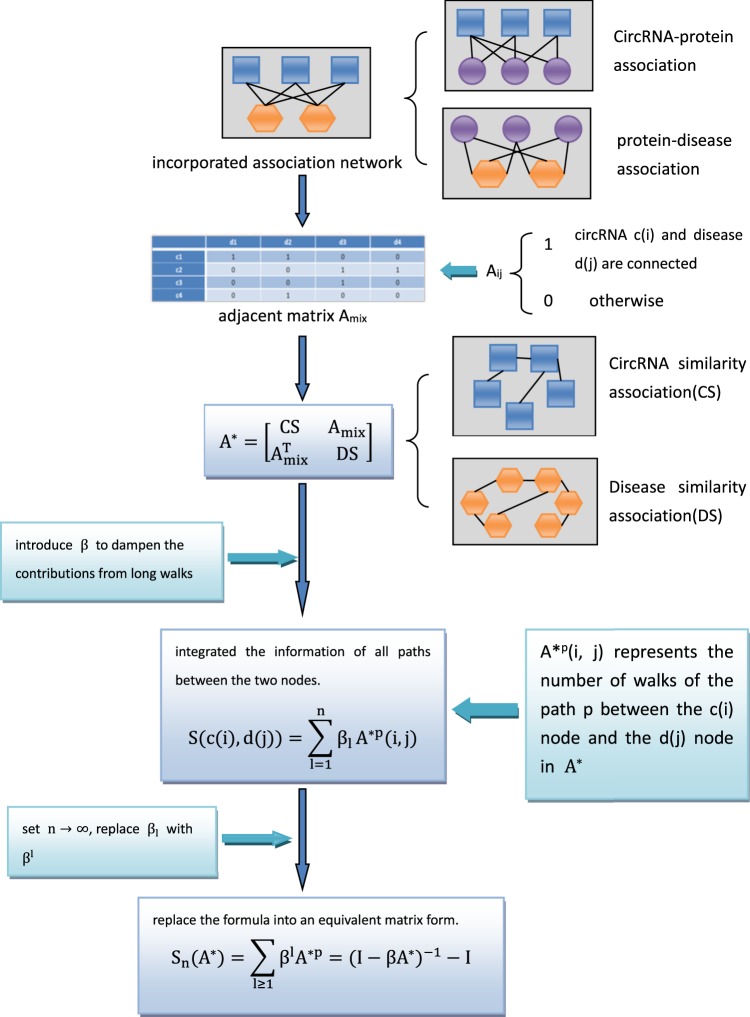
Flowchart of the KATZCPDA method.

Fig. 6 shows a flowchart of KATZCPDA. Heterogeneous data sources were used to construct three interaction/similarity networks (circRNA, disease, and protein) and three different association networks (circRNA-protein, protein-disease, and circRNA-disease). We then generated an incorporated circRNA-disease association network by integrating these three interlinked networks. The network can be represented as an undirected graph *G*_*i*_ = (*V*_*i*_, *E*_*i*_), where *V*_*i*_ is the set of nodes and *E*_*i*_ is the set of undirected edges. Each node in the network represents a biological entity (circRNA, protein, or disease), and each undirected edge represents a relationship, similarity, or interaction between the connected objects.

We assumed that *W* was the adjacency matrix of network *G*. We normalized *W* to obtain *W*′ according to the topological information of the network using the normalization formula ${\rm{W}}^{\prime} ={{\rm{D}}}_{{\rm{G}}}^{-1/2}{{\rm{WD}}}_{{\rm{G}}}^{-1/2}$, where the diagonal matrix *D*_*G*_ is defined that *D*_*G*_*(i, i)* is the sum of the *i*^*th*^ values of *W*, namely, ${{\rm{D}}}_{{\rm{G}}}({\rm{i}},\,{\rm{i}})=\sum _{{\rm{j}}}{{\rm{W}}}_{{\rm{i}},{\rm{j}}}$, and *W*′ is the symmetric matrix calculated by the formula ${\rm{W}}^{\prime} ({\rm{i}},\,{\rm{j}})={\rm{W}}({\rm{i}},\,{\rm{j}})/\sqrt{{\rm{D}}({\rm{i}},\,{\rm{i}}){\rm{D}}({\rm{j}},\,{\rm{j}})}$.

The primary focus of this study is the identification of circRNA-disease association pairs. Thus, we calculated the number of walks and lengths of the path from the circRNA node c(i) to the disease node d(j). A^p^(i, j) represents the number of walks in the path p from the c(i) node to the d(j) node, and the length of the walks in p is 1. The integration of the information about all the paths between two nodes can provide information on the potential association between nodes c(i) and d(j). In this approach, the contribution of the length of the walks to the prediction association probability is inversely proportional on every walk, that is, a shorter walking length l of path p between the nodes means a higher similarity between them. The introduction of the nonnegative coefficient sequence β_1_ (if the walks of length l_1_ are shorter than l_2_, β_11_ is larger than β_12_) dampens the contributions from long walks. Therefore, the potential association between circRNA and disease can be predicted using the following formula:

$${\rm{S}}({\rm{c}}({\rm{i}}),\,{\rm{d}}({\rm{j}}))=\sum _{{\rm{l}}=1}^{{\rm{n}}}{{\rm{\beta }}}_{{\rm{l}}}{{\rm{A}}}^{{\rm{p}}}({\rm{i}},\,{\rm{j}})$$

Because the constructed network is suitable for inclusion in the adjacency matrix, the formula was introduced into an equivalent matrix form. We further set ${\rm{n}}\to \infty$ and thus ${{\rm{\beta }}}_{{\rm{l}}}\to 0$. Therefore, β_1_ was replaced by β^1^, A^P^(i,j) was replaced by A^l^, and the following formula was obtained:

$${{\rm{S}}}_{{\rm{n}}}({\rm{A}})=\sum _{{\rm{l}}\ge 1}\,{{\rm{\beta }}}^{{\rm{l}}}{{\rm{A}}}^{{\rm{l}}}={({\rm{I}}-{\rm{\beta }}A)}^{-1}-{\rm{I}}$$

where matrix S_n_(A) contains the similarity scores for all circRNAs and all diseases. A higher score indicates a stronger association between the circRNA and disease.

The integration of the adjacency matrix A_mix_ corresponding to the incorporated circRNA-disease association network G_mix_ with the circRNA similarity matrix CS and the disease similarity matrix DS yielded the matrix A*, which provides all the information for the entity. The method then ultimately obtains S_n_(A*) as the prediction. The integrated matrix denotes as followed:

$${{\rm{A}}}^{\ast }=[\begin{array}{cc}{\rm{CS}} & {{\rm{A}}}_{{\rm{mix}}}\\ {{\rm{A}}}_{{\rm{mix}}}^{{\rm{T}}} & {\rm{SD}}\end{array}]$$
